# Importance of HER2 Work-Up and Treatment Even in Patients with Poor Performance Status: A Case Report

**DOI:** 10.1155/2014/731581

**Published:** 2014-04-13

**Authors:** Ali Suner, Hakan Buyukhatipoglu, Ozan Balakan, Mehmet Emin Kalender, Turgay Ulas, Alper Sevinc, Celalettin Camci

**Affiliations:** ^1^Department of Medical Oncology, School of Medicine, Gaziantep University, Kilis Yolu Uzeri, 27310 Gaziantep, Turkey; ^2^Depatment of Internal Medicine, Harran University School of Medicine, Sanliurfa, Turkey

## Abstract

Gastric cancer is one of most common types of cancers. Metastatic gastric cancer has a poor prognosis and is accepted as incurable at this stage. Treatment of metastatic gastric cancer did not progress substantially until new targeted agents have come out. Recently published ToGA trial showed promising results in HER2 overexpressing metastatic gastric cancer. In this case we present a case with an excellent complete response with anti-HER2 treatment. Most importantly, we wanted to emphasize (1) the importance of early determination of HER2 overexpression, and (2) to draw attention of anti-HER2 agents in the first line treatment even in patients with a poor performance status.

## 1. Introduction

In USA, through the year 2014 approximately 22000 new gastric cancers and 11000 gastric cancer-related deaths have been expected [1]. In the past 10 years a significant decreasing trend has been observed in gastric cancer incidence and mortality rates especially in US and European countries [2]. The most important prognostic factor is tumor stage in gastric cancer; however, most of the patients are diagnosed either in inoperable locally advanced stage or in metastatic stage [3, 4]. The 5-year overall survival is 5 to 20%, and median survival time is less than 1 year in these groups [5]. Meta-analyses of several randomized trials demonstrated that combination treatments yielded better results than single agent chemotherapy and best supportive care [6]. While fluoropyrimidine based regimens—either or not combined with platinum—have been widely used in first step treatment, taxanes and irinotecan have been used in later steps [7]. Even combination treatments benefit and prognosis of advanced stage gastric and gastroesophageal cancers are still not good; hence less toxic new therapies are needed.

Human epidermal growth factor receptor 2 (HER2, CerbB2) is a well-defined target for new treatments. It is overexpressed in 7 to 34% of gastric cancers [8]. The benefit of trastuzumab in advanced gastric or gastroesophageal cancer was addressed in trastuzumab for gastric cancer (ToGA) trial. According to ToGA trial the overall survival benefit was 2.7 months (11.1 months in chemotherapy arm and 13.8 months in chemotherapy + trastuzumab arm), which was statistically significant [9]. In this paper we present a case of metastatic gastric cancer that showed excellent response under trastuzumab, despite poor prognostic factors and the advanced stage he had.

## 2. Case Report

**Figure 1:**
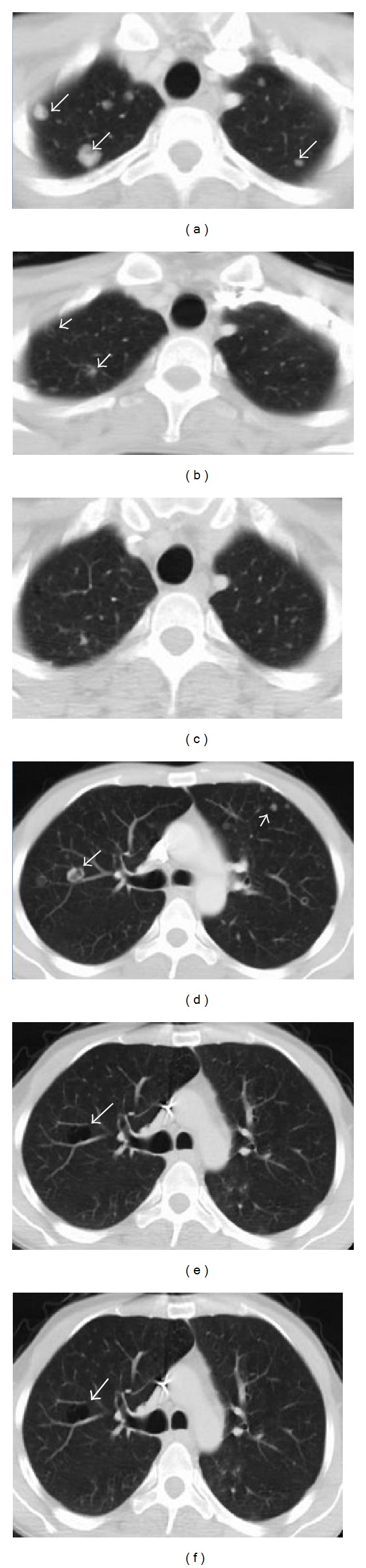
In different sections (above and bottom) regressing of metastatic nodules in the lungs ((a) and (d), at onset; (b) and (e), October; (c) and (f), December); note the cavitations in (e) and (f).

**Figure 2:**
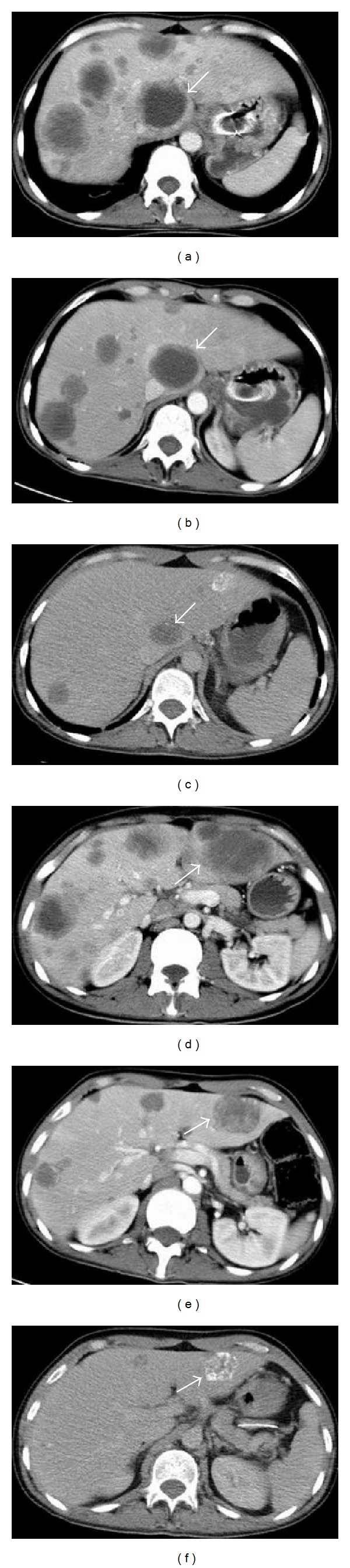
In different sections (above and bottom) regressing of metastatic lesions in the liver ((a) and (d), at onset; (b) and (e), October; (c) and (f), December); note the calcifications in (c) and (f).

**Figure 3:**
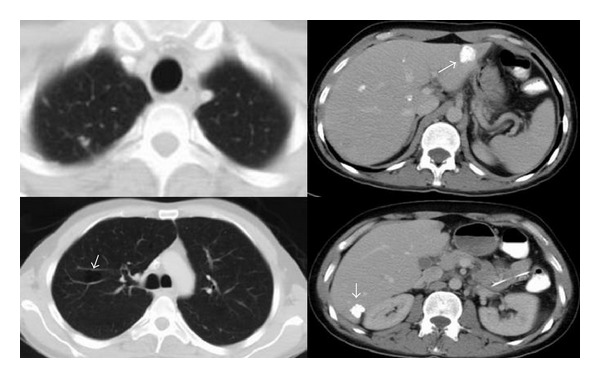
Complete response in July 2013 in the lungs and liver.

A 35-year-old male patient was referred to our medical oncology department with a gastric tumor that surrounded the gastric cardia leading to partial obstruction. Endoscopic biopsy result was adenocarcinoma. After screening for staging, the patient was found to have multiple lung and hepatic metastases (Figures 1(a) and 1(d); Figures 2(a) and 2(d)). According to Eastern cooperative oncology group (ECOG) his performance status was 3. His pathology specimens that were reevaluated for HER2 overexpression were both positive with immunohistochemistry and fish staining. Therefore, we offered him first-line chemotherapy with docetaxel, carboplatin, and trastuzumab (TCH) in August 2012. After 2 cycles of TCH we achieved partial response (October 2012) (Figures 1(b) and 1(e); Figures 2(b) and 2(e)). After 6 cycles we achieved further partial response (December 2012) (Figures 1(c) and 1(f); Figures 2(c) and 2(f)). However, he developed grade 3 neuropathy after these cycles. Therefore, we substituted docetaxel with 5-Fluorouracil (5-FU). He was given 6 more cycles of trastuzumab and carboplatin with 5-FU. After these 6 cycles we achieved complete response in both liver and lung metastases (April 2013). The clinical appearance of complete response was rather unusual which we have not seen before in our past clinical experience, with cavitations on lungs and calcifications on liver (Figure 3). On endoscopy on April 2013 complete response was achieved for the primary lesion (primary tumor located in cardia). Beginning with May 2013 the patient was placed on capecitabine and trastuzumab. On the last follow-up visit on July 2013 (Figure 3) complete response has been achieved. He was doing well with no symptoms and his ECOG performance status was 0.

## 3. Discussion

In Western countries about two thirds of gastric cancer patients are diagnosed with locally advanced or metastatic disease. Median survival time for these patients is around 10 months. Poor performance status (PS > 2), liver metastases, peritoneal metastases, and increased alkaline phosphatase levels are considered unfavorable prognostic factors [10].

The new trend in cancer management has been shifted to targeted biologic agents, which are less toxic and more tolerable and may provide survival benefit either with or without chemotherapy depending on the cancer type and histology. Gastric cancer is one of the new therapeutic fields for targeted agents. Antiepidermal growth factor receptor treatment has come out as a new promise [11]. ToGA is the first phase 3 randomized study evaluating the effects of anti-Her2 treatment in advanced gastric cancer. As we mentioned above results of ToGA trial are promising [9].

Isolated complete responses have been reported in some clinical trials with very low ratios (0%–0.7%) with traditional chemotherapy in metastatic or inoperable locally advanced gastric cancer [12, 13]. However, in ToGA study, complete response rate was reported as 5% with trastuzumab containing treatments [9]. In our case, the patient had multiple liver and lung metastases. He demonstrated an excellent response to trastuzumab containing protocols. We achieved a survival time more than it would be expected in similar stage gastric cancer patients with multiple metastasis and poor performance status.

We want to report this case for several reasons. First, this excellent response is worth reporting because it is very unusual to achieve complete response in a patient in whom the best supportive care might be reasonable. Second, when the tumor completely regressed the remaining calcification in liver and cavitations in lungs were very unusual, which we have not seen before. Finally, the most important point is the concept that some patients may demonstrate excellent responses to new biological agents. In summary, we believe that pathological work-up for HER2 status should not be ignored even in patients with poor performance status.
